# Intimate partner violence against reproductive age women during COVID-19 pandemic in northern Ethiopia 2020: a community-based cross-sectional study

**DOI:** 10.1186/s12978-020-01002-w

**Published:** 2020-10-07

**Authors:** Gebremeskel Tukue Gebrewahd, Gebreamlak Gebremedhn Gebremeskel, Degena Bahrey Tadesse

**Affiliations:** 1 Department of Emergency Medicine and Critical Care Nursing, School of Nursing, Aksum University, Aksum, Ethiopia; 2 Department of Adult Health Nursing, School of Nursing, Aksum University, Aksum, Ethiopia

**Keywords:** COVID-19, Violence, Women and Ethiopia

## Abstract

**Background:**

As the global pandemic of corona virus (COVID-19) spreads across continents and communities, people are forced to respond with strict preventive measures such as staying at home and keeping social distance. In relation with these measures, particularly with the staying at home, increasing rates of domestic violence are beginning to surface. Hence, this study was aimed at determining the prevalence of intimate partner violence against reproductive age women in northern Ethiopia during the COVID-19 pandemic.

**Methods:**

A community-based cross-sectional study design was employed. The data were collected during the period of April to May, 2020 using interviews and a self-administered standard questionnaire. The data were entered into the Epi-data manager version 4.2 and exported to SPSS 22 for analysis. The descriptive analysis such as frequency distribution, percentage, and measures of central tendency were used. This was followed by binary and multiple logistic regression analysis to infer the association between the outcome and independent variables.

**Results:**

A total of 682 participants were included in the study. The prevalence of intimate partner violence against women was found to stood at 24.6% with psychological violence being the most prevalent (13.3%), followed by physical (8.3%) and sexual violence (5.3%). Women were more likely to suffer from violence if they were housewives (AOR, 95% CI (18.062 (10.088, 32.342))), age less than 30 (AOR, 95% CI (23.045 (5.627, 94.377))), women with arrange marriage (AOR, 95% CI (2.535 (1.572, 4.087))) and women with husband’s age being “between” 31–40 (AOR, CI 95% (2.212 (1.024, 4.777))).

**Conclusions:**

This study showed the presence of a relatively high prevalence of intimate partner violence against women. Thus, public reporting of any cases or concerns of abuse is critical and vital to mitigate the problem.

## Plain English summary

Intimate partner violence against reproductive age women is an existing occurrence in the human biosphere. Any violence can exert a negative impact on the women’s physical, psychological, sexual and reproductive health. This study assessed the prevalence of intimate partner violence and contributing factors against reproductive age women in northern Ethiopia using a community-based cross-sectional study design.

The data was collected from a Kebele (administrative unit) of the community to obtain information of the selected 682 women. A written consent was obtained for each woman and they were interviewed individually. The data collectors and supervisors were well trained. The interviewed data were categorized and analyzed to identify predictors of intimate partners violence. Around one-fifth of the study participants suffered violence from their intimate spouse or friend. Being a housewife, having younger age, having arranged marriage, and having younger-aged husbands were the most significant predictors of women intimate violence. Thus, devotion is required to track and report any sort of women violence in the era of COVID-19 pandemic.

## Background

Violence against women (VAW) is recognized as a significant public health problem in both developed and developing worlds. Besides violating human rights, it has grave consequences on women’s physical, mental, sexual, and reproductive health. Intimate partner violence against women (IPVAW) is defined by the World Health Organization (WHO) as women’s self-reported experience of all forms of violence [1–3].

An article published by WHO reported that 1 in 3 (35%) of women worldwide have experienced either physical or sexual violence by intimate partner or ex-partner in their lifetime. The most prevalent is violence by an intimate partner with around one-third (30%) of the women reporting that they have suffered numbers of physical and/ or sexual violence by their intimate partner [4].

During the COVID-19, increasing rates of domestic violence are beginning to surface around the world. Notably, in china, domestic violence has tripled during the stay-at-home order issued by the country. The universal trend of reports on the increasing domestic violence cases is likely to continue throughout the pandemic and may only represent a “tip of the iceberg” as many victims still find themselves trapped with the perpetrator and unable to report the abuse [5]. The United States issued a warning on intimate partner violence due to increasing novel virus corona 2019. Conditions like stress confinement, financial uncertainty and a desire to control the disaster may hump up the risk of IPV. Current lockdown declaration in China, Spain, and Italy is increasing IPV call emergency. In several areas of the UK, France, Alberta call reports have increased by 20, 30% and 30–50%, respectively. In Ontario, a regional police’s report constitutes a 22% cases of domestic violence and sexual assault [6].

In Australia, since the issuance of stay-at-home order came into effect, domestic violence was reported to be increased by 75%. Similar problem ranging from 21 to 35% and 32–36% happened in the United States and France, respectively, following social isolation and quarantine. The problem is occurring over the entire world and rumors of domestic violence and family abuse around the globe have been inflamed since the implementation of social distance and lockdown order [7].

Despite the WHO reports and recommendations to reduce violence against women, the COVID-19 pandemic may increase incurring specific challenges for women in our community and hence needs an integrated approach in addressing the root cause of the challenge. Thus, this study was aimed at assessing the prevalence of IPV against reproductive age women and its contributing factors during COVID-19 in northern Ethiopia.

## Methods

### Study setting and period

The study was carried out in Aksum town, northern Ethiopia. The town is found in the Central Zone of Tigray region, a state found 1025 km north of Addis Ababa. It has a total population of 60,706, of which 30,991 (51.0%) are females and 29,775 (49.0%) are males. Administratively, the town is divided into five Kebles [7]. The study was conducted between April to May, 2020. The sources population of the study were all reproductive age women in Aksum town.

### Sample size and sampling technique

Sample size was determined using single population proportion formula with the assumption that 28.1% of women had physical violence [8]. At 5% marginal error (d), and the addition of 10% for none response, as well as by multiplying the sample size by 2 to account for design effect of the sampling, the final total sample size was found to be 682. There are 5 kebeles (lowest administrative unit) in Aksum city. The Kebeles are similar to each other in several aspects, so selecting one from each Kebele using the lottery method was suffice for representation. A systematic random sampling technique with an interval at every K^th^ ($\frac{\mathrm{study}\ \mathrm{population}\ }{\mathrm{desired}\ \mathrm{sample}\ \mathrm{size}\ }$) was used to select the study subject. Every 6th house hold women participated and random start was made by lottery method. In cases where there were two respondents in one household, one of them was selected with simple random technique.

### Data collection tools and procedures

A validated structured questionnaire, adopted from WHO core questionnaire on domestic intimate partner violence [8], was prepared in the local language (Tigrigna) and used to interview selected reproductive age women who were requested for verbal and written consent to participate in the study. They were interviewed individually and the interview time was after lunch time and before evening. Their partners were not participating in the interview to keep confidentiality and allow the women to freely explain the mishappennings and to minimize conflict with their partners. If any member of the family was nearby, the interview time was shifted to another period. The interview consisted of socio-demographic profiles of the women, various acts of gender-based violence, husband’s behavior, and about their degree of power in deciding family-related issues. Three diploma holder female nurses were recruited for data collection and one BSc holder was recruited as supervisor. The overall data collection process was coordinated and overseen by the principal investigator.

### Data quality control

A questionnaire which was prepared in English was translated in Tigrigna and back to English to check its consistency. It was pre-tested on 5% of women in one kebele outside the study area. After the pretest, the questionnaire was modified as necessary. Data collection was carried out by trained female nurses from other units of the health facilities. The collected data were checked for completeness daily by the supervisor and the investigators monitored the overall quality of data collection process.

### Study variables

Dependent variables:

Violence against women (psychological, physical and sexual violence).

Independent variable.

Socio-demographic factor:

**Maternal factors**; Age, sex, marital status, family size, level of education, occupation, religion, ethnicity and marriage duration.

**Husband factor**; age, behavior, monthly income, ethnicity, religious belief, educational status and occupation.

**Family- related factors:** Type of marriage, number of children (alive), and annual household income.

### Operational definitions

Physical violence was considered to have happened if either of these 6 acts happened: slapped / thrown object that could hurt, pushed/ shoved, hit with a fist/ something else that could hurt, kicked/dragged, choked/ burnt on purpose, and threatened to use/ actually used a gun, knife, or other weapon against the women. Sexual violence was measured using three acts (physically forced to have sexual intercourse against willing, had sexual intercourse bearing a fear of what partner might do, and sexually degraded or humiliated) [2]. The current prevalence of intimate partner violence was defined as the proportion of reproductive age women who experienced one or more acts of these violence by an intimate partner or ex-partner.

### Data processing and analysis

Data were coded, entered, edited, and cleaned by Epi-data manager version 4.2 and then exported into SPSS version 22 for analysis. The data were analyzed descriptively using frequency distribution, percentage and measure of central tendency. Binary logistic regression model was used to infer the association between the outcome and independent variables. In the bi-variate analysis, variables with *P*-value < 0.25 were included in the multivariable binary logistic regression. Odds ratio with 95% confidence level was computed and *p*-value < 0.05 was described as having a significant association. The result was presented using texts, charts and tables.

## Results

### Socio-demographic characteristics

The mean age of the respondents was 29.78 years (± 5.78SD) with a range of 22 years. The smallest age was 21 years. More than half (52.9%) of the respondents had low family income while a third (34.6%) of the women had no formal education. More than two-thirds (68.6%) of the women were housewives. Most of the participants (68.3%) were married with arrange marriage and live together currently while the remaining (31.7%) had a love marriage. Of the respondents, few (5.3%) had no children. The majority (62.8%) of the women had husbands with an alcohol drinking habit while a sizable proportion (40.2%) of the participants had husbands with aggressive behavior (Table 1).

**Table 1:** Socio-demographic and socioeconomic characteristics of reproductive age women, Aksum town, 2020 (*n* = 682)

	Numbers percentage	
**Women’s age**		
Less than 30	431	63.2
31–40	215	31.5
Above 40	36	5.3
**Ethnicity**		
Tigray	675	99
Amara	7	1.0
**Religion**		
Orthodox	624	91.5
Muslim	58	8.5
**Women’s level of education**		
Uneducated	236	34.6
Primary	183	26.8
Secondary	191	28.0
College or university	72	10.6
**Women’s occupation**		
House wife	468	68.6
Employed	214	31.4
**Types of marriage**		
Arranged	466	68.3
Love marriage	216	31.7
**Number of lives children**		
No child	36	5.3
1–2	360	52.8
3–4	251	36.8
5+	35	5.1
**Husband’s age**		
Less than 30	72	10.6
31–40	360	52.8
Above 40	250	36.7
**Husband’s level of education**		
Uneducated	175	25.7
Primary	148	21.7
Secondary	168	24.6
College or university	191	28.0
**Family monthly income**		
Low	361	52.9
Medium	213	31.2
High	108	15.8
**Does your husband drink alcohol?**		
No	254	37.2
Yes	428	62.8
**Is your husband aggressive?**		
No	408	59.8
Yes	274	40.2

**Fig. 1:**
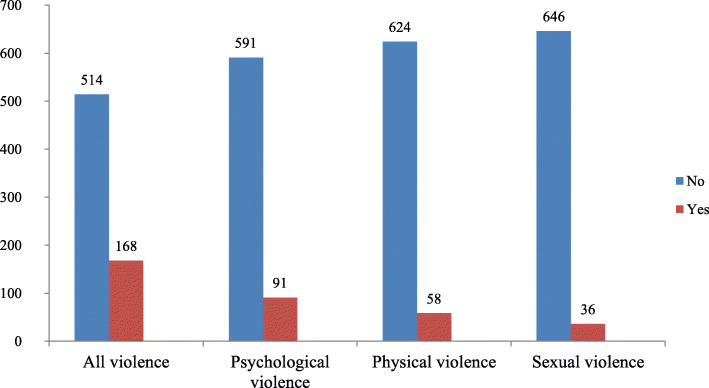
Prevalence of the psychological (13.3%), physical (8.3%) and sexual violence (5.3%)

The result on violence against women was obtained using thirteen WHO questions regarding psychological, physical and sexual violence. Each of the participant’s response on violence was dichotomously coded (yes, no) to verify in amount whether or not they have experienced violence from their intimate partners (Fig. 1).

Psychological violence was the predominant type of violence (13.3%) which resulted mostly (90.2%) by way of insulting or made feel bad about oneself while the least common (1.6%) way of the violence was scaring or intimidating on purpose. 8.3% of the participants were suffering from physical violence, and 4.3% of this violence was through slapping or objects being thrown at them. None (0%) of the participants experienced physical violence by threatening to use or actually using a gun, knife or other weapon. Sexual violence was experienced by 5.3% of the participating subjects with 3.5% of these subjects suffering from having sexual intercourse under duress. However, none of the participants reported to have a forced sexual act that they found to be degrading or humiliating (Table 2).

**Table 2:** Types of intimate violence on reproductive age women during COVID-19 pandemic, Aksum town, 2020 (*n* = 682)

Violence type	Question items	Frequency (%)
**Psychological violence**	Has he insulted you or made you feel bad about yourself?	
	No	615 (90.2)
	Yes	67 (9.8)
	Has he belittled or humiliated you in front of other people?	
	No	638 (93.5)
	Yes	44 (6.5)
	Has he done things to scare or intimidate you on purpose?	
	No	671 (98.4)
	Yes	11 (1.6)
	Has he threatened to hurt you or someone you care about?	
	No	632 (92.7)
	Yes	50 (7.3)
**Physical violence**	Has he slapped you or thrown something at you that could hurt you?	
	No	653 (95.7)
	Yes	29 (4.3)
	`Has he pushed or shoved you?	
	No	657 (96.3)
	Yes	25 (3.7)
	Has he hit you with his fist or with something else that could hurt you?	
	No	673 (98.7)
	Yes	9 (1.3)
	Has he kicked you, dragged you or beaten you up?	
	No	681 (99.9)
	Yes	1 (.1)
	Has he choked or burnt you on purpose?	
	No	680 (99.7)
	Yes	2 (.3)
	Has he threatened to use or actually used a gun, knife or other weapon against you?	
	No	682 (100.0)
	Yes	
**Sexual violence**	Has he physically forced you to have sexual intercourse when you didn’t want to?	
	No	670 (98.2)
	Yes	12 (1.8)
	Did you ever have sexual intercourse when you didn’t want because you were afraid of what he might do?	
	No	658 (96.5)
	Yes	24 (3.5)
	Has he forced you to do something sexual that you found degrading or humiliating?	
	No	682 (100.0)
	Yes	

The frequency distribution of the prevalence of violence against women during COVID-19 lockdown was measured using thirteen questions and by giving a numerical value for each question (1 = Yes (correct), 0 = No (incorrect). Four questions were used for psychological violence, six questions for physical violence, and three questions for sexual violence (Table 2).

In bivariate analysis, the independent variables that showed association with the outcome variable were women’s age, types of marriage, women’s levels of education, women’s occupation, husband’s levels of education and husband’s behavior. After considering all assumptions of binary logistic regression, those variables which had *p*-value<=0.25 at bi-variable analysis entered into multivariable logistic regression. After controlling for confounding effect women’s age; level of education, occupation, types of marriage and husband’s age had significant association with intimate partner violence against women in the multi variant logistic regression model (Table 3).

**Table 3:** Factors associated with prevalence of intimate partner violence against reproductive age, Aksum town, 2020 (*n* = 682)

Variables		IPVAW		COR		95% C.I. for EXP(B)		AOR		95% C.I. for EXP(B)	
		No (%)	Yes (%)	Sig	Exp(B)	Lower	Upper	Sig	Exp(B)	Lower	Upper
**Women’s age**											
Less than 30		335 (65.2%)	96 (57.1%)	.063	1.972	.963	4.040	.000 **	23.045	5.627	94.377
31–40		156 (30.4%)	59 (35.1%)	.148	1.320	.906	1.922	.000 **	3.690	1.939	7.023
Above 40		23 (4.5%)	13 (7.7%)					1			
**Women’s level of education**											
Uneducated		176 (34.2%)	60 (35.7%)	.007	3.166	1.376	7.281	.011 **	.450	.244	.832
Primary		133 (25.9%)	50 (29.8%)	.004	3.491	1.500	8.125	.627	.860	.469	1.579
Secondary		140 (27.2%)	51 (30.4%)	.005	3.383	1.456	7.859	.526	.744	.298	1.856
College or university		65 (12.6%)	7 (4.2%)					1			
**Women’s occupation**											
Housewife		109 (21.2%)	105 (62.5%)	.000	.161	.111	.235	.000 **	18.062	10.088	32.342
Employed		405 (78.8%)	63 (37.5%)					1			
**Types of marriage**											
Arranged		145 (28.2%)	71 (42.2%)	.001	.537	.374	.771	.000 **	2.535	1.572	4.087
Love marriage		369 (71.8%)	97 (57.7%)					1			
**Husband’s age**											
Less than 30		47 (9.1%)	25 (14.9%)	.939	.985	.673	1.442	.727	.827	.284	2.406
31–40		276 (53.7%)	84 (50.0%)	.060	1.722	.977	3.033	.043 **	2.212	1.024	4.777
Above 40		191 (37.2%)	59 (35.1%)					1			
**Husband behavior**											
Drink alcohol	No	182 (35.4%)	72 (42.9%)					1			
	Yes	332 (64.6%)	96 (57.1%)	.084	.731	.512	1.042	.247	.774	.501	1.194
Aggressive	No	318 (61.9%)	90 (53.6%)					1			
	Yes	196 (38.1%)	78 (46.4%)	.057	1.406	.989	1.999	.239	1.313	.834	2.066
**Husband’s levels of education**											
Uneducated		113 (21.9%)	56 (33.3%)	.547	.846	.491	.596	.150	.544	.237	1.247
Primary		247 (48%)	73 (43.4%)	.014	1.677	1.109	.150	.507	1.241	.656	2.345
Secondary		84 (16.3%)	21 (12.5%)	.638	.870	.487	.507	.596	.830	.417	1.653
College or university		70 (13.6)	18 (10.9%)					1			

**Key**: **- Significant, if COR- crud odd ratio (95% CI, *p* < 0.25) and AOR-adjusted odd ratio (95% CI, *p* < 0.05)

1 – Reference

## Discussions

This study attempted to assess the prevalence of intimate partner violence against reproductive age women during the COVID-19 pandemic among the society of Aksum town. In this study, socio-demographic and violence-related responses of 682 study participants were considered.

According to this study, 13.3% (CI. 11–16) experienced psychological violence and 8.3% (CI. 6.6–10.6) experienced physical violence while sexual violence was reported in 5.3% (CI. 3.7–6.9) of study subjects.

In this study, 24.6% (95% CI, 21.4, 28) women reported to have suffered all types of violence during the COVID-19 Pandemic. This is higher than a study conducted on violence against women by their husband or friend in the UK [6]. This variance might be due to deference in the time of the study as well as the socio-demographic difference of the study populations. On the other hand, our study’s result in the prevalence of the violence was in line with a study conducted in Ontario [6] and United States [7]. This may be due to the similar population characteristics in responding to psychological or financial disturbance while staying home.

Our finding’s on the prevalence of violence was smaller in magnitude than a study done in Alberta [6]. Since the stay-at-home order came into effect, domestic violence to women by their spouses has increased in Australia [9] and large numbers of victims of psychological, physical and sexual violence are reported to be taking place in Mexico [10]. In Brazil and Italy, domestic violence is reported to have jumped up. Similarly, in Spain, reports of a horrendous domestic violence-related homicide – a manner that is unfortunately likely to continue around the world have been reported [5]. This may be due to the long period of spending time in home which may restrict relaxation, which may lead to stress, as well as causing conflicting decision power in parenthood administration.

Our finding was congruent with Germany’s report that that the hasty spread of the virus in the absence of battered therapy or a vaccine is forcing countries to respond with strong preventive measures such as social distancing, commanding schools, business closures, and impressive travel restrictions to reduce the transmission of the infectious disease. However, the resulting accumulation of frustration, anger and severe depression may speed up the domestic violence especially with spouses or ex-partners [11].

Intimate partner violence against women is already the most common grieving report worldwide. Even in the absence of community health emergency, a study conducted in Afghanistan on the experience of psychological, physical and/or sexual violence indicated a prevalence of 11.8 and 23.1%, respectively, while a prevalence of 15% was reported in Thailand [12, 13] . Our study’s result was larger than these studies, and this may be due to the difference in the study time and study area. In southwest Ethiopia, the life-time prevalence of IPV of physical or sexual or both was 64.7% [2], and in southern brazil 56% reported to have experienced the problem [3]. Similarly, in Iran, the prevalence of physical, sexual and emotional violence was 16.4, 18.6 and 44.4%, respectively [14], while IPV studies in Zimbabwe and Ethiopia showed 15, 30%, respectively [15, 16]. In contrast to our study; these articles are studied over a longer period and this may have led to higher prevalence of IPV. On the other hand, a study conducted on IPV in Brazil on psychological violence indicated violence to have happened in 25.3%, while physical and sexual violence reported in 9.9 and 5.7% of participants [17]. Almost similar report was found in our study.

Housewives were 18.062 times more likely to suffer from violence than those who were employed, and women younger than thirty years old were 23.045 times most likely to face violence. This result was consistent with a study conducted in Zimbabwe [16]. Unlike these results, the levels of education of women’s and their husband’s were the most significant risk factors for violence in Iran [14].

## Conclusions

This study showed a relatively higher prevalence of violence against women. Husbands’ level of education, being a housewife, and marrying with arrange marriage were highly related with domestic violence by their respective husbands. Identifying the high-risk individuals is important to strengthen the link between social and national health system, family laws as well as police investigations to prevent the high impact of violence against women.

### Limitations

This study needs peer-based information about the real experience of the damage on women since the study subjects have not felt at ease to express their response freely. Hence, the outcome may have been subjected to recall bias. Moreover, the timing of this study may have flared up the findings during the COVID era relative with pre-COVID era.

## Data Availability

The data sets used and analyzed during the current study are presented within the manuscript and available from the corresponding author upon reasonable request.

## Abbreviations

COVID-19: Corona Virus Disease in 2019
WHO: World Health Organization
IPVAW: Intimate Partner Violence Against Women
DHS: Demographic and Health Survey
